# Essential items for reporting of scaling studies of health interventions (SUCCEED): protocol for a systematic review and Delphi process

**DOI:** 10.1186/s13643-019-1258-3

**Published:** 2020-01-11

**Authors:** Amédé Gogovor, Hervé Tchala Vignon Zomahoun, Ali Ben Charif, Robert K. D. McLean, David Moher, Andrew Milat, Luke Wolfenden, Karina Prévost, Emmanuelle Aubin, Paula Rochon, Giraud Ekanmian, Jasmine Sawadogo, Nathalie Rheault, France Légaré

**Affiliations:** 1 Health and Social Services Systems, Knowledge Translation and Implementation component of the Quebec SPOR-SUPPORT Unit, Quebec, Canada; 2 Tier 1 Canada Research Chair in Shared Decision Making and Knowledge Translation, Université Laval, Quebec, Canada; 3 Department of Family Medicine and Emergency Medicine, Université Laval, Quebec, Canada; 4 Centre de recherche sur les soins et les services de première ligne de l’Université Laval, Pavillon Landry-Poulin, 2525, Chemin de la Canardière, Quebec, QC G1J 0A4 Canada; 5 International Development Research Centre, PO BOX 8500, Ottawa, Ontario K1G 3H9 Canada; 6 Faculty of Medicine and Health Sciences, Stellenbosch University, Francie van Zijl Drive, Tygerberg, 7505 South Africa; 7 School of Epidemiology and Public Health, University Research Chair in Systematic Reviews, Ottawa, Canada; 8 Ottawa Methods Centre, Ottawa Hospital Research Institute, The Ottawa Hospital, General Campus, Centre for Practice Changing Research Building, 501 Smyth Road, PO BOX 201B, Ottawa, Ontario K1H 8L6 Canada; 9 Centre for Epidemiology, NSW Ministry of Health, Australia, LMB 961, North Sydney, 2059 Australia; 10 School of Public Health, Faculty of Medicine and Health, University of Sydney, Edward Ford Building (A27) Fisher Road, Sydney, NSW 2006 Australia; 11 School of Medicine and Public Health, University of Newcastle, Locked Bag 10, Wallsend, NSW 2287 Australia; 12 Patient partner, Quebec, Canada; 13 Women’s College Research Institute, Women’s College Hospital, University of Toronto, 76 Grenville Street, Toronto, Ontario M5S 1B2 Canada

**Keywords:** Scaling, Spread, Reporting guideline, Systematic review, Delphi method

## Abstract

**Background:**

The lack of a reporting guideline for scaling of evidence-based practices (EBPs) studies has prompted the registration of the Standards for reporting studies assessing the impact of scaling strategies of EBPs (SUCCEED) with EQUATOR Network. The development of SUCCEED will be guided by the following main steps recommended for developing health research reporting guidelines.

**Methods:**

***Executive Committee.*** We established a committee composed of members of the core research team and of an advisory group.

***Systematic review***. The protocol was registered with the Open Science Framework on 29 November 2019 (https://osf.io/vcwfx/). We will include reporting guidelines or other reports that may include items relevant to studies assessing the impact of scaling strategies. We will search the following electronic databases: EMBASE, PsycINFO, Cochrane Library, CINAHL, Web of Science, from inception. In addition, we will systematically search websites of EQUATOR and other relevant organizations. Experts in the field of reporting guidelines will also be contacted. Study selection and data extraction will be conducted independently by two reviewers. A narrative analysis will be conducted to compile a list of items for the Delphi exercise. ***Consensus process****.* We will invite panelists with expertise in: development of relevant reporting guidelines, methodologists, content experts, patient/member of the public, implementers, journal editors, and funders. We anticipated that three rounds of web-based Delphi consensus will be needed for an acceptable degree of agreement. We will use a 9-point scale (1 = extremely irrelevant to 9 = extremely relevant). Participants’ response will be categorized as irrelevant (1–3), equivocal (4–6) and relevant (7–9). For each item, the consensus is reached if at least 80% of the participants’ votes fall within the same category. The list of items from the final round will be discussed at face-to-face consensus meeting. ***Guideline validation****.* Participants will be authors of scaling studies. We will collect quantitative (questionnaire) and qualitative (semi-structured interview) data. Descriptive analyses will be conducted on quantitative data and constant comparative techniques on qualitative data.

**Discussion:**

Essential items for reporting scaling studies will contribute to better reporting of scaling studies and facilitate the transparency and scaling of evidence-based health interventions.

## Background

The scaling of evidence-based practices (EBPs) can be considered as one of the ultimate phases of knowledge translation. Whereas “knowledge translation” in general is concerned with the conversion of research into action, “scaling” is how we optimize the magnitude, variety, equity, and sustainability of research-informed actions. Among the diverse concepts used in knowledge translation and implementation science and defined elsewhere [1] such as adoption, adaptation, dissemination, spread, and sustainability, scaling is “often used in the context of international, national, and regional health programs” [2]. The concept of scaling is relatively new in the health sector [3]. Scaling EBPs emerged from the World Health Organization’s (WHO) strategic approach to strengthening reproductive health policies and programs, mainly in low- and middle-income countries where scaling up strategies were implemented in different areas of health [4, 5]. In high-income countries, scaling of EBPs is now gaining more and more interest. Years ago, Bégin et al. even referred to Canada as “a country of perpetual pilot projects” because proven projects or outcomes of pilot projects are rarely moved into stable, funded programs and or transferred across jurisdictions [6]. As pointed by a family doctor and advocate for public health care in Canada, “it’s time to build systems that support the implementation of large-scale change” [7]. Similar statements about the lack of scaling up of EBPs were made in other countries [8]. Reasons include governments’ inclination for short-term results, the lack of expertise in scaling science in high-income countries, and the fact that no one in our health care system holds that responsibility [6, 7]. Scaling up is defined as “deliberate efforts to increase the impact of successfully tested health innovations so as to benefit more people and to foster policy and program development on a lasting basis” [5]. Other variants of scaling include scaling out, deep, and down. Our view is to be inclusive of all types of, and approaches to, scaling EBPs. We believe we will learn more about scaling and its effective reporting, with an open and accepting approach to contextualized language and models. What matters most in our view are the potential benefits or impacts of scaling. And there is evidence that scaling of EBPs may promote benefits such as equitable access to quality care and prevent waste of time, resources, and energy [5, 6, 9].

Findings from studies assessing the impact of scaling strategies in health care need to be reported adequately so their results can facilitate their replication and be translated in policy. However, deficiencies in the quality of reporting of health research are well documented in the literature [10–14]. According to Hoffmann et al., up to 60% of interventions in a sample of trial reports were inadequately described [15]. A systematic review on scaling up strategies of EBPs in primary care noted vast inconsistencies in how authors reported their results, with none reporting all the needed information for assessing scalability of EBPs or the effectiveness of scaling up strategies [10]. Consequences of inadequate reporting include lapses of scientific integrity (e.g., failure to honor research participants’ accounts or measured data, fairness) [16], difficulty to judge the reliability and robustness of the results, and the relevance of the evidence [13].

To remedy this situation, the EQUATOR (Enhancing the QUAlity and Transparency Of health Research) Network was created to improve the quality of publications by providing resources and training relating to the reporting of health research and by assisting in the development, dissemination, and implementation of reporting guidelines [17]. A reporting guideline can be defined as “a checklist, flow diagram, or explicit text to guide authors in reporting a specific type of research, developed using explicit methodology” [13]. In the developing science of scaling, the rare systematic reviews of scaling up EBPs commented on the poor quality of reporting [10, 12]. This may be attributed to the lack of reporting guidelines relevant to the process of scaling.

A few reporting guidelines in the field of implementation science have been developed recently. These include the Standards for Reporting Implementation Studies (StaRI) [18] and the reporting guidelines for implementation and operational research [19]. StaRI is a reporting guideline for phase IV implementation studies and does not cover the core components of scaling up strategies [10]. The reporting guidelines for implementation and operational research, developed by WHO, are broad and covered the field of implementation rather than specific methods or study designs [19].

**Fig. 1:**
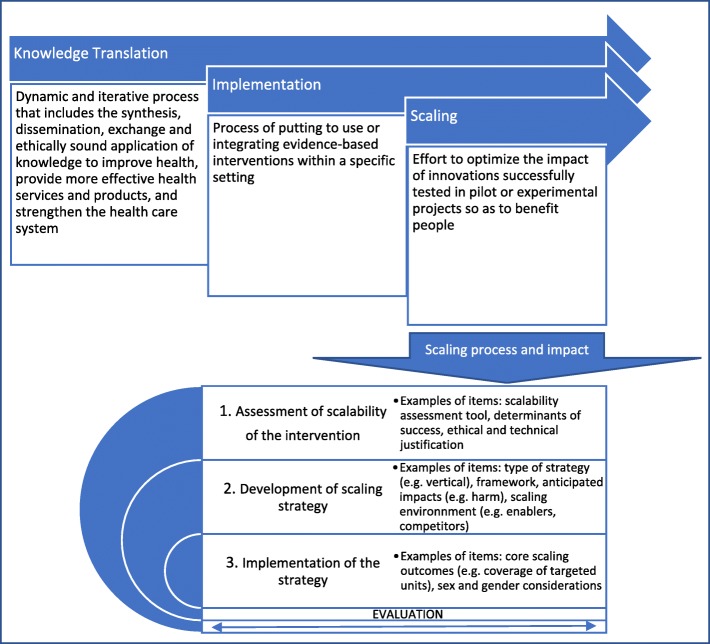
SUCCEED standards for reporting studies assessing scaling process and impact. Content adapted from [1, 5, 10, 12, 21, 22]

The lack of a specific reporting guideline for scaling studies and the identification of several gaps [10] has prompted the registration of the standards for reporting trials assessing the impact of scaling up strategies of EBPs (SUCCEED) with EQUATOR [3]. These gaps include (a) poor description of scaling strategies, (b) lack of mention of the type of scaling strategy (e.g., vertical, horizontal), (c) unclear distinction between the EBP and the strategies used to scale the EBP, and (d) inconsistent reporting (e.g., no information on assessing the scalability of the EBPs, lack of a clear measure of the scaling outcome). Our goal for proposing the new reporting guideline is to help address these gaps in reporting and knowledge translation related to the scaling of EBPs, including lack of assessment of potential harms, little information on sex and gender issues, and absence of patients and public engagement in designing the scaling strategies [3, 20]. Figure 1 depicts the place of scaling in the context of knowledge translation and the incremental contribution of SUCCEED reporting guideline. SUCCEED will be informed by elements addressed in existing reporting guidelines such as the clear distinction between the implementation strategies and the intervention being implemented in StaRI. Examples of items that will be specific to our reporting guideline include (a) description of the scalability assessment of the EBP in the introduction, (b) ethical and technical justification of the scaling, (c) justification of the scaling unit, (d) description of stakeholders, and (e) sex and gender considerations (objectives, measures of outcomes and effects, analyses, discussion).

This project is embedded in a peer-reviewed and 7-year Canadian Institutes for Health Research funded Foundation grant titled: “Scaling up shared decision making for patient-centered care” [23]. In addition, the project will also contribute to the science of reporting guidelines as it will be among the first to integrate sex and gender considerations. The rationale of taking into account sex and gender in the development of the reporting guideline stems from several elements: while their importance in the manifestation and management of health conditions and in health outcomes is now getting better established, their considerations are rarely integrated in research design and reporting guidelines [24, 25]; importance of appropriate use of the terms sex and gender based on documentation that they are often misused, misunderstood, confused, or conflated in health research [26], unlike other health determinants such as education, employment, and income; and fulfillment of the role of a reporting guideline that is to help reduce waste in health research by addressing the deficiency in the quality of its reporting and better inform practice, policy, and programs. Moreover, the success of implementation and scaling is highly context-dependent, particularly for complex interventions. Thus, sex and gender considerations will be integrated in the literature review and in the development of the guideline (e.g., mention of appropriate use of sex and gender, extent to which both sexes were represented in the panel group and in each trial, presentation of disaggregated data). We will include a few items in our reporting guideline, for example, (a) the stakeholders involved in the process of scaling have to be described according to their sex/gender, (b) all outcomes have to be reported by sex/gender, and (c) data analyses have to be sex/gender-based. Addressing these gaps will contribute to knowledge translation and implementation and scaling science. In turn, this should contribute to improving health outcomes and equity.

## Objectives

The aim of this project is to develop the SUCCEED, a reporting guideline for studies assessing scaling strategies.

To accomplish this, specific objectives are follows: (1) establish an executive committee that will oversee the development process of the guideline, (2) review the literature to document current reporting and identify relevant items for a reporting guideline for studies assessing the impact of scaling strategies, (3) prioritize items for a reporting guideline for studies assessing the impact of scaling strategies using a Delphi process and/or a consensus meeting, (4) pilot test the new reporting guideline SUCCEED, and (5) develop a comprehensive dissemination plan.

## Methods

The development of SUCCEED is guided by the steps recommended for developing health research reporting guidelines and available on the EQUATOR Network website [13, 27]. The five main steps corresponding to the specific objectives are described as follows.

### Executive committee

An executive committee composed of members of the core research team and of an advisory group is established to oversee the development process of the guideline. Members of the core research team are Amédé Gogovor, postdoctoral fellow and co-principal investigator; France Légaré, Tier 1 Canada Research Chair in Implementation of Shared Decision Making in Primary Care and registrant of SUCCEED supervisor and co-principal investigator; David Moher, co-supervisor with extensive expertise in systematic review and reporting guideline development; Hervé Zomahoun, scientific coordinator of Knowledge Translation component of the Québec SPOR SUPPORT Unit, with extensive expertise in Cochrane systematic review methodology; and Ali Ben Charif, postdoctoral fellow with an expertise in scaling up in primary care and first author of the systematic review on effective strategies for scaling up EBPs in primary care [10]. The advisory group is composed of:

*-*Content experts in scaling up and implementation science: Andrew J. Milat, Luke Wolfenden, and Robert McLean (implementation science in low- and middle-income countries and representative of a funding agency)

*-*Patient and the public representatives: Emmanuelle Aubin and Karina Prévost (at least two, as per SPOR-SUPPORT Unit guidelines)

-Expert in sex and gender: Paula Rochon.

### Literature review

This includes the documentation of the quality of reporting scaling interventions and identification of relevant items for SUCCEED.

#### Evidence of poor reporting

To inform the quality of reporting in scaling studies, we will conduct a secondary analysis of the articles included in the previous systematic review of our team [10]. A list of key elements of scaling up will be compiled using reference documents in scaling up (e.g., Milat et al., WHO-ExpandNet) and validated by scaling experts. We will report the proportion of the articles that did not report these key elements. The deficiencies identified will be considered for inclusion in SUCCEED.

#### Items for a reporting guideline for scaling up studies: a systematic review

An initial step in the development of this reporting guideline is to systematically compile a list of potential items [13].

##### Inclusion and exclusion criteria

We will apply the following criteria presented in Table 1.

**Table 1:** Inclusion and exclusion criteria

Inclusion	
Reporting standard	Any guide or document that provides instructions or recommendations, e.g., reporting guideline, checklist, guidance, framework, standard
Type of studies	Scaling [defined as “effort to increase the impact of innovations successfully tested in pilot or experimental projects so as to benefit more people”] or implementation (science) [defined as “scientific study of the use of strategies to adopt and integrate evidence-based health interventions into clinical and community settings to improve patient outcomes and benefit population health”]
Domain	Health
Type of document	Any
Timeline	No restrictions
Language	No restrictions
Exclusion	Documents for formatting guidance by journal editors and publishers such as “Instructions to Authors” [38]

##### Search strategies

A search strategy will be developed by our information specialist for MEDLINE followed by an iterative process of revision by members of the research team and validation by a second information specialist using a Peer Review of Electronic Search Strategies tool [28] (see Additional file 1 for a sample of MEDLINE search strategy). A combination of free (keywords) and controlled (e.g., MeSH) vocabularies will be performed: e.g. standard*, guidance, framework, reporting guideline*, checklist*, requirement*, instruction*, publishing, good practice*, implementation, implementation science, scaling up, scaling out, scale up, spread. The search strategy will be then translated into the following electronic databases: EMBASE, PsycINFO, Cochrane Library (Methodology Register), CINAHL, and Web of Science, from inception. No language restriction will be applied. In addition, we will systematically search websites of relevant organizations (e.g., EQUATOR Network, WHO/ExpandNet, Canadian Foundation for Healthcare Improvement (CFHI), International Development Research Centre, Australia NSW Government, Global Reporting Initiative, additional relevant organizations) using the Canadian agency for Drugs and Technologies in Health (CADTH)’s Grey Matters checklist [29]. Experts in the field of reporting guidelines will also be contacted.

##### Data management

EndNote will be used to remove the duplicates, and the resulting unique records will be exported to an Internet-based system (Covidence) for the selection. We will use Microsoft Excel to record the data extraction.

##### Study selection

Two reviewers will independently screen for titles and abstracts and select eligible studies, after pilot testing the eligibility criteria on a randomly selected sample of records. Discrepancies will be resolved by consensus or by a third reviewer if necessary.

##### Data extraction

We will develop an extraction form informed by the Cochrane Checklist of items to consider in data collection [30] and three guidelines [5, 18, 21]. The form will include (1) general characteristics (e.g., title, short name, corresponding author name and contact information, number and type of items of the checklist, dimensions covered, presence of a flow diagram); (2) elements of the development process (e.g., methods for initial items, consensus methods used); (3) elements (items possibly relevant to) of implementation/scaling strategies and outcomes (e.g., type of strategy, coverage, fidelity); (4) description on integration of sex and gender: we will extract the presence of any of sex- and gender-related words in the checklist/main or explanation text (e.g., sex, gender, male, female); and (5) other information (e.g., funding source, conflict of interest). The form will be tested on a 10% random sample of the included studies for data collection. We will contact the authors of the included documents to request relevant missing information.

##### Quality appraisal

We will develop a list of criteria to assess the validity of retrieved documents based on expert consultation. Examples of criteria include number and type of stakeholder groups involved, use of a consensus process, and pilot test [Moher D., personal communication]. Two reviewers will independently grade (yes, no, unclear) the quality.

##### Analysis

A narrative analysis will be conducted. We will summarize the data using descriptive statistics (e.g., frequencies, percentages). A list of items will be generated and divided into the following categories: title, abstract, introduction (background, aim), methods (e.g., theoretical framework, core components, and assessment of scaling potential of the EBP), results (e.g., effectiveness of EBP, quantitative metrics of scaling success, cost, fidelity, sustainability), discussion (e.g., implications for practice and policy), and other information (funding source and conflict of interest). We will use the Preferred Reporting Items for Systematic Reviews and Meta-Analyses (PRISMA) statement [31] to report the review and document any important protocol amendments.

### Consensus process

This step will include two phases: a series of online questionnaires (e-Delphi) and a face-to-face meeting.

#### e-Delphi

The study will use the Delphi technique, a series of sequential surveys, interspersed by controlled feedback. The method is widely used in health care settings to gain consensus of opinion of a group of experts [32–34]. It will be conducted and reported using the guidance on Conducting and Reporting Delphi Studies [35].

##### Recruitment of experts

Panelists will be selected to capture the multiple perspectives of those that influence the design, implementation, evaluation, and reporting of scaling of health interventions. A list of expert panelists will be compiled by the research team and include authors of the articles included in the literature review; authors of relevant reporting guidelines; methodologists (experts in systematic review and reporting guideline development); content experts (healthcare professionals and scaling up experts); patient and the public representatives; implementers, e.g., CFHI, The Evidence Project; editors from journals that publish to implementation science and scaling up and from varied countries including low- and middle-income countries, e.g., Implementation Science, Bull. World Health Organization, PloS One, Am J Trop Med Hyg; and funders, e.g., FRQS, CIHR, NIH, EU, WHO, IDRC, Grand Challenges Canada, Melinda and Bill Gates, charities that fund primary care research. An invitation will be sent to all the identified panelists, and an active list and a backup list will be compiled based on their response and availability to participate in the e-Delphi and/or the face-to-face meeting. All the invitees will be asked to indicate their willingness to participate in the evaluation of the guideline and in a semi-structured interview. Prior to the start process, we will assess any conflict of interest among the members of the research team.

##### Procedure

We anticipated that three rounds of web-based Delphi consensus will be needed for an acceptable degree of agreement; if not, a final round will be undertaken. Summaries of previous rounds will be compiled for subsequent rounds. We will use the REDCap [36] platform to administer the survey. The full questionnaire will be pre-tested prior to administration.

-First survey round: The survey will start with general questions (including country of employment, sex, disciplinary field, and years of experience) and will continue with the list of items of relevance of SUCCEED.

-Subsequent rounds: a new list of items (items that do not reach consensus, new suggested items) will be presented along with scores from the previous round.

##### Reaching agreement

We will use the traditional 9-point scale (1 = extremely irrelevant to 9 = extremely relevant) [35]. Participants’ response will be categorized as irrelevant (1–3), equivocal (4–6), and relevant (7–9). For each item, the consensus is reached if at least 80% of the participants’ votes fall within the same category (1–3, 4–6, or 7–9) [33–35, 37]. The questionnaire will include a free-text box for the panelists to provide comments or suggest new items. Items that are rated as equivocal and new suggested items will be listed in subsequent rounds until the final round.

#### Face-to-face consensus meeting

The objectives of the meeting are to (a) produce the final list of items for SUCCEED reporting guideline, (b) discuss strategy for producing the documents of the reporting guideline and their dissemination, and (c) distribute the post-meeting tasks such as draft of the guideline documents, obtention of endorsement, and website development [13]. Steps to produce the final list of items are as follows: (i) present the results of Delphi exercise (name, rationale, and score of each item); (ii) discuss the rationale and relevance for including the items in the checklist; and (iii) vote on non-consensual items. We will invite around 20 expert panelists for 1.5 to 2 days meeting. We will record all the sessions and use note-taking services to report the discussions. At the end of the meeting, the final list of items for SUCCEED reporting guideline will be defined.

### Guideline validation

#### Study design

To pilot test the SUCCEED checklist, we will use cross-sectional and qualitative approaches.

#### Participants

All the authors of the identified studies in our previous systematic review [10] and additional studies identified by updating the searches will be invited (less than 50 studies are expected).

#### Data collection

We will collect general characteristics of the participants (e.g., country, sex, field of expertise). For the quantitative component, participants will be asked to use the SUCCEED checklist to report their study and provide comments on the items. A brief semi-structured interview of 15–30 min on the form (layout, wording, and structure) and barriers and facilitators of using the guideline will be conducted with each participant. The interviews will be conducted in person, by telephone or video conference (e.g., GoToWebinar), recorded, and transcribed verbatim.

#### Analyses

Descriptive analyses will be conducted on quantitative data: number and percentages of items reported, interview data, and comments will be analyzed using constant comparative techniques and thematically synthetized by one researcher and validated by the other members of the research team. The results will inform how the guideline improve the quality of reporting and provide information and examples to enrich the elaboration of the statement of the SUCCEED and the accompanied explanatory paper.

## Ethical considerations

Ethical approval will be obtained from the Centre intégré universitaire de santé et de services sociaux de la Capitale-Nationale (CIUSSS-CN) Ethics Board. Oral, electronic, and written inform consent will be obtained from all the participants of the e-Delphi, the consensus meeting, and the pilot study.

## Discussion

Essential items for reporting scaling studies will contribute to better reporting of scaling studies and facilitate the transparency and scaling of evidence-based health interventions. The dissemination of this reporting guideline will start with the publication of the protocol of the development of SUCCEED. The publication strategy will be finalized, building on the discussion from the consensus meeting. The development of the guideline will be reported in a statement document that will include the rationale, a brief description of the meeting and participants involved, and the checklist of SUCCEED. Contact will be made with journal editors to secure multiple and simultaneous publication of the guideline and related editorials. The active dissemination approaches will include presenting at relevant scientific conferences, holding webinars, and workshops. We will develop a website (hosted by our institution) and set up a Twitter® account for ongoing interactions with users and will explore other social media platforms as this initiative grows. Finally, we will use different indicators to assess the use of the guideline. These include analytic metrics of the website, the number of “retweets” and “likes,” and the number of new publications that used the guideline. Other methods including pre-post or stepped wedge designs may be used.

## Supplementary information

**Additional file 1.** Sample MEDLINE search strategy

## Data Availability

The datasets used and/or analyzed during the current study are available from the corresponding author on reasonable request.
