# Supplementary material for: Essential items for reporting of scaling studies of health interventions (SUCCEED): protocol for a systematic review and Delphi process
Source: Syst Rev. 2020 Jan 11;9:11. doi: 10.1186/s13643-019-1258-3 (PMC6954577; doi:10.1186/s13643-019-1258-3)
Supplement: Supplementary file 1 — Additional file 1. Sample MEDLINE search strategy [file 13643_2019_1258_MOESM1_ESM.docx]

**Additional file 1**

Sample MEDLINE search strategy

| **Number** | **Search strategy keywords** | **Search results** |
| --- | --- | --- |
| 1 | exp Writing/st [Standards] | 2 797 |
| 2 | (((((good or best) adj2 practi#e*) or guide or guides or guideline* or checklist* or "check list*" or framework* or standard* or recommend* or guidance* or requirement* or instruct* or consensus or "aide memoir*" or quality or "worked example*" or criteri* or critique* or design or designs or clarity or statement* or parameter* or advice* or policy or policies or accura* or appropriate* or "minimum information" or tool* or complete or protocol* or advice* or uniform* or better or strengthen* or transparen*) adj2 (report* or writ*)) or "research standard*" or "publishing standard*" ).ti.  or (((((good or best) adj2 practi#e*) or guide or guides or guideline* or checklist* or "check list*" or framework* or standard* or recommend* or guidance* or requirement* or instruct* or consensus or "aide memoir*" or quality or "worked example*" or criteri* or critique* or design or designs or clarity or statement* or parameter* or advice* or policy or policies or accura* or appropriate* or "minimum information" or tool* or complete or protocol* or advice* or uniform* or better or strengthen* or transparen*) adj2 (report* or writ*)) or "research standard*" or "publishing standard*").ab.  or ( ((((good or best) adj2 practi#e*) or guide or guides or guideline* or checklist* or "check list*" or framework* or standard* or recommend* or guidance* or requirement* or instruct* or consensus or "aide memoir*" or quality or "worked example*" or criteri* or critique* or design or designs or clarity or statement* or parameter* or advice* or policy or policies or accura* or appropriate* or "minimum information" or tool* or complete or protocol* or advice* or uniform* or better or strengthen* or transparen*) adj2 (report* or writ*)) or "research standard*" or "publishing standard*").kf. | 67 164 |
| 3 | 1 or 2 | 69 466 |
| 4 | (("scaling" or widespread or spread$ or spreading or "rolling out" or "roll out" or "rolls out" or "rolled out" or "scale$ up" or "scale$ out" or upscaling or scalability or scalable) adj5 (innovation$ or intervention$ or technolog* or practice* or care or initiative* or program* or product* or therap* or service*)).ti.  or (("scaling" or widespread or spread$ or spreading or "rolling out" or "roll out" or "rolls out" or "rolled out" or "scale$ up" or "scale$ out" or upscaling or scalability or scalable) adj5 (innovation$ or intervention$ or technolog* or practice* or care or initiative* or program* or product* or therap* or service*)).ab.  or (("scaling" or widespread or spread$ or spreading or "rolling out" or "roll out" or "rolls out" or "rolled out" or "scale$ up" or "scale$ out" or upscaling or scalability or scalable) adj5 (innovation$ or intervention$ or technolog* or practice* or care or initiative* or program* or product* or therap* or service*)).kf | 22 017 |
| 5 | ((bring* or brought or taking or take* or increas* or going or implement* or econom*) adj5 scal* adj5 (innovation$ or intervention$ or technolog* or practice* or care or initiative* or program* or product* or therap* or service*)).ti.  or ((bring* or brought or taking or take* or increas* or going or implement* or econom*) adj5 scal* adj5 (innovation$ or intervention$ or technolog* or practice* or care or initiative* or program* or product* or therap* or service*)).ab.  or ((bring* or brought or taking or take* or increas* or going or implement* or econom*) adj5 scal* adj5 (innovation$ or intervention$ or technolog* or practice* or care or initiative* or program* or product* or therap* or service*)).kf. | 2 123 |
| 6 | ("reverse innovation*" or "trickle-up innovation*").ti. or ("reverse innovation*" or "trickle-up innovation*").ab. or ( "reverse innovation*" or "trickle-up innovation*" ).kf | 40 |
| 7 | (transfer* adj5 (innovation$ or intervention$ or technolog* or initiative*)).ti. or (transfer* adj5 (innovation$ or intervention$ or technolog* or initiative*)).ab. or (transfer* adj5 (innovation$ or intervention$ or technolog* or initiative*)).kf. | 4 787 |
|  |  |  |
| 8 | 4 or 5 or 6 or 7 | 28 469 |
|  |  |  |
| 9 | Translational medical research/ or Information Dissemination/ or exp "diffusion of innovation"/ or exp Knowledge Management/ | 42 399 |
| 10 | (("knowledge to action" or "continuing education" or "know-do" or implement* or dissemin* or diffus* or adopt* or adapt*) adj5 (research or science or bioscience or biomedic* or innovation* or intervention* or technolog* or practice* or care or initiative* or program* or product* or therap* or service*)).ti.  or (("knowledge to action" or "continuing education" or "know-do" or implement* or dissemin* or diffus* or adopt* or adapt*) adj5 (research or science or bioscience or biomedic* or innovation* or intervention* or technolog* or practice* or care or initiative* or program* or product* or therap* or service*)).ab.  or (("knowledge to action" or "continuing education" or "know-do" or implement* or dissemin* or diffus* or adopt* or adapt*) adj5 (research or science or bioscience or biomedic* or innovation* or intervention* or technolog* or practice* or care or initiative* or program* or product* or therap* or service*)).kf. | 176 598 |
| 11 | ((applied or mediation* or translat*) adj5 (research or science or bioscience or biomedic*)).ti.  or ((applied or mediation* or translat*) adj5 (research or science or bioscience or biomedic*)).ab.  or ((applied or mediation* or translat*) adj5 (research or science or bioscience or biomedic*) ).kf | 30 458 |
| 12 | ((mediation* or translat* or populari#ation or populari#e or populari#ed or populari#ing) adj5 (innovation* or intervention* or technolog* or practice* or care or initiative* or program* or product* or therap* or service*)).ti.  or ((mediation* or translat* or populari#ation or populari#e or populari#ed or populari#ing) adj5 (innovation* or intervention* or technolog* or practice* or care or initiative* or program* or product* or therap* or service*)).ab.  or ((mediation* or translat* or populari#ation or populari#e or populari#ed or populari#ing) adj5 (innovation* or intervention* or technolog* or practice* or care or initiative* or program* or product* or therap* or service*)).kf. | 26 806 |
| 13 | (knowledge) adj3 (transfer* or translat* or broker* or mobil* or uptake or "up take" or adapt* or dispers* or exchange* or application or utili#ation or utili#e or utili#ed or utili#ing or communicat* or cycle? or transform* or action? or manage*)) adj5 (innovation* or intervention* or technolog* or practice* or care or initiative* or program* or product* or therap* or service*)).ti.  or (knowledge adj3 (transfer* or translat* or broker* or mobile* or uptake or "up take" or adapt* or dispers* or exchange* or application or utili#ation or communicat* or cycle? or transform* or action? or manage*) adj5 (innovation* or intervention* or technolog* or practice* or care or initiative* or program* or product* or therap* or service*)).ab.  or (knowledge adj3 (transfer* or translat* or broker* or mobile* or uptake or "up take" or adapt* or dispers* or exchange* or application or utili#ation or communicat* or cycle? or transform* or action? or manage*) adj5 (innovation* or intervention* or technolog* or practice* or care or initiative* or program* or product* or therap* or service*)).kf. | 4 265 |
| 14 | 10 or 11 or 12 or 13 | 228 168 |
| 15 | 9 or 14 | 260 622 |
| 16 | 8 or 15 | 283 800 |
| 17 | 3 and 16 | 3 410 |
| 18 | exp Animals/ NOT exp Humans/ | 4 576 104 |
| 19 | 17 NOT 18 | 3 370 |
